# Interleukin-27 Gene Therapy Prevents the Development of Autoimmune Encephalomyelitis but Fails to Attenuate Established Inflammation due to the Expansion of CD11b^+^Gr-1^+^ Myeloid Cells

**DOI:** 10.3389/fimmu.2018.00873

**Published:** 2018-04-24

**Authors:** Jianmin Zhu, Jin-Qing Liu, Zhihao Liu, Lisha Wu, Min Shi, Jianchao Zhang, Jonathan P. Davis, Xue-Feng Bai

**Affiliations:** ^1^Pediatric Translational Medicine Institute, Shanghai Children’s Medical Center, School of Medicine, Shanghai Jiao Tong University, Shanghai, China; ^2^Department of Pathology and Comprehensive Cancer Center, Ohio State University, Columbus, OH, United States; ^3^Department of Physiology, Ohio State University, Columbus, OH, United States

**Keywords:** interleukin-27, IL-30, experimental autoimmune encephalomyelitis, Th1, Th17, PD-L1, Treg cells, central nervous system

## Abstract

Interleukin-27 (IL-27) and its subunit P28 (also known as IL-30) have been shown to inhibit autoimmunity and have been suggested as potential immunotherapeutic for autoimmune diseases such as multiple sclerosis (MS). However, the potential of IL-27 and IL-30 as immunotherapeutic, and their mechanisms of action have not been fully understood. In this study, we evaluated the efficacy of adeno-associated viral vector (AAV)-delivered IL-27 (AAV-IL-27) and IL-30 (AAV-IL-30) in a murine model of MS. We found that one single administration of AAV-IL-27, but not AAV-IL-30 completely blocked the development of experimental autoimmune encephalomyelitis (EAE). AAV-IL-27 administration reduced the frequencies of Th17, Treg, and GM-CSF-producing CD4^+^ T cells and induced T cell expression of IFN-γ, IL-10, and PD-L1. However, experiments involving IL-10-deficient mice and PD-1 blockade revealed that AAV-IL-27-induced IL-10 and PD-L1 expression were not required for the prevention of EAE development. Surprisingly, neither AAV-IL-27 nor AAV-IL-30 treatment inhibited EAE development and Th17 responses when given at disease onset. We found that mice with established EAE had significant expansion of CD11b^+^Gr-1^+^ cells, and AAV-IL-27 treatment further expanded these cells and induced their expression of Th17-promoting cytokines such as IL-6. Adoptive transfer of AAV-IL-27-expanded CD11b^+^Gr-1^+^ cells enhanced EAE development. Thus, expansion of CD11b^+^Gr-1^+^ cells provides an explanation for the resistance to IL-27 therapy in mice with established disease.

## Introduction

Interleukin-27 (IL-27) is an IL-12 family of cytokines that is composed of Epstein–Barr virus-induced gene 3 (EBI3) and IL-27p28 (also known as IL-30) subunits. Produced by activated antigen-presenting cells (APCs) such as dendritic cells and macrophages (1–3), IL-27 signals through a heterodimeric receptor (IL-27R) consisting of the WSX-1 and the gp130 subunits, which is expressed in a variety of cell types including T lymphocytes and myeloid cells (4). IL-27 has been shown to inhibit Th17 responses (5, 6), and induce IL-10 (7–9) and PD-L1 (10) expression in T cells, and has been shown to inhibit inflammation in animal models of autoimmune diseases (11, 12), including animal models of multiple sclerosis (MS) (9, 13, 14) and collagen-induced arthritis (15). These results suggest that IL-27 may be a potential immunotherapeutic for human autoimmune diseases.

Indeed, previous studies (9, 13) have revealed that IL-27 delivered systemically can inhibit the development of experimental autoimmune encephalomyelitis (EAE) in mice, an experimental model of MS. However, systemic injection of IL-27 is costly, and it is also difficult to maintain an effective concentration in the circulation. In this context, gene therapy could serve as an effective alternative approach. For instance, IL-30 gene therapy has been shown to efficiently inhibit autoimmune inflammation in the central nervous system (CNS) and eye (16), and lentiviral IL-27 gene delivery to the CNS inhibits neuroinflammation (17). Adeno-associated viral vectors (AAVs) are highly efficient delivery agents for gene therapy (18). AAV vectors can efficiently transfer genes of interest to a broad range of mammalian cell types leading to high levels of stable and long-term expression after a single application (19). AAV vectors are also known to have low immunogenicity and have been used in human clinical trials (20–22). In this study, we have evaluated the therapeutic efficacy of AAV-delivered IL-27 (AAV-IL-27) and IL-30 (AAV-IL-30) in T cell-mediated autoimmune encephalomyelitis, where the inflammation in the CNS is considered to be mediated mainly by Th17/Th1 responses and T cells producing GM-CSF (23, 24). We found that one single administration of AAV-IL-27, but not AAV-IL-30 completely prevented EAE development. Experiments involving IL-10-deficient mice and PD-1 blockade revealed that AAV-IL-27-induced IL-10 and PD-L1 expression were not required for the inhibition of EAE development. However, neither AAV-IL-27 nor AAV-IL-30 treatment inhibited EAE development and Th17 responses when given at disease onset. We found that mice with established EAE had significant expansion of CD11b^+^Gr-1^+^ myeloid cells, and AAV-IL-27 treatment further expanded these cells and induced their expression of multiple cytokines including Th17-promoting cytokines such as IL-6 and IL-23. Adoptive transfer of AAV-IL-27-expanded CD11b^+^Gr-1^+^ cells enhanced EAE development. Thus, systemic delivery of IL-27 can efficiently prevent EAE development and the priming of Th17 responses. However, the therapeutic potential of IL-27 is limited by its failure in inhibiting ongoing EAE, and shutting down established Th17 responses, presumably due to the expansion of CD11b^+^Gr-1^+^ myeloid cells.

## Materials and Methods

### Mice

C57BL/6, C57BL/6 mice with targeted mutation of the IL-27Rα (IL-27Rα^−/−^) and IL-10 (IL-10^−/−^) genes were purchased from the Jackson Laboratory (Bar Harbor, ME, USA). 2D2 TCR transgenic mice (25) were described before (26, 27). All mice were maintained in the animal facilities of The Ohio State University, and the studies were approved by the Institutional Animal Care and Use Committee.

### Induction and Assessment of EAE

C57BL6, IL-10^−/−^, and 2D2 mice of 8–12 weeks of age were immunized subcutaneously with 200 µg MOG 35-55 emulsified in PBS:CFA (1:1) in a total volume of 100 µL. MOG35-55 (MEVGWYRSPFSRVVHLYRNGK) was purchased from Genemed Synthesis, Inc. (South San Francisco, CA, USA). The purity of the peptide was greater than 90%. Mice also received 150 ng of pertussis toxin (List Biological, Campbell, CA, USA) in 200 µL PBS *via* the tail vein immediately after the immunization and again 48 h later. The mice were observed every day for the development of EAE symptoms using parameters as we described before (26, 27).

### Production of AAV Viruses and Mice Treatment

Adeno-associated viral vector-IL-27, AAV-IL-30, and AAV-ctrl viruses were produced as we previously described (28). Briefly, IL-27 or IL-30 cDNA were inserted into an AAV carrier vector under the control of the CMV-chicken beta-actin hybrid promoter (29, 30). The IL-27 or IL-30 carrier AAV vector was compacted with a helper vector in 293K cells into the AAV serotype 8 (AAV8), which could achieve high expression in muscles (31, 32). AAV viruses were injected into mice intramuscularly (i.m.) using a dose of 2 × 10^11^ DRP/mouse diluted in 50 µL PBS.

### ELISA

Serum samples were collected from mice treated with AAV-IL-27, AAV-IL-30, and AAV-ctrl viruses at various time points after viral injection. The presence of IL-27 or IL-30 in serum was detected using ELISA kits purchased from eBiosciences (IL-27) or R&D systems, Inc. (IL-30).

### Isolation of Mononuclear Cells From Spinal Cords

Spinal cord tissues from AAV-IL-27, AAV-ctrl virus-treated or -untreated mice with EAE were removed and cut into about 2-mm pieces and incubated in 10 mM Hepes/NaOH buffer containing 1 mg/mL of collagenase IV (Sigma, St. Louis, MO, USA) for 1 h at 37°C. Then, the tissues were dispersed with syringe, filtered through a 100-mm wire mesh, and centrifuged at 2,000 rpm for 5 min at 4°C. After centrifugation, tissue pallets were re-suspended in 15 mL 30% Percoll (Pharmacia, Uppsala, Sweden), then centrifuged against 70% Percoll in a 50-mL tube for 15 min. The cell monolayer at the 30–70% Percoll interface was collected and washed once for further staining and flow cytometry analyses.

### Antibodies and Flow Cytometry

FITC-, PE-, APC-, or Percp-labeled antibodies to CD4 (GK1.4), CD11b (M1/70), CD45 (30-F11), Gr-1 (RB6-8C5), Ly6C (AL-21), IL-6 (MP5-32C11), IL-10 (JES5-2A5), IL-17 (TC11-18H10), IFN-γ (XMG1.2), GM-CSF (MP1-22E9), FoxP3 (NRRF-30), PD-L1 (MIH5), IL-27Rα (2918), and isotype control antibodies were purchased from BD Biosciences (San Diego, CA, USA). Procedures for cell surface marker staining and intracellular cytokine staining were the same as we described (26, 27). Briefly, for staining of cell surface markers, mononuclear cells from spleens, lymph nodes, and CNS were stained with various antibodies in staining buffer (PBS with 1% FCS) and incubated on ice for 30 min. After washing with staining buffer, cells were fixed in 1% paraformaldehyde in PBS. For intracellular cytokine staining, cells were stimulated in culture medium for 4 h with 100 ng/mL of phorbol 12-myristate 13-acetate and 500 ng/mL of ionomycin in the presence of Golgi^stop^ (1:1,500; BD Biosciences). Viable cells were then fixed in IC fixation buffer (eBioscience), permeabilized with 1× permeabilization buffer (eBiosciences), and stained with respective antibodies. Foxp3 staining was performed according to the manufacturer’s protocol (BD Biosciences). Cells were collected on a FACSCalibur flow cytometer, and data were analyzed using the FlowJo software (Tree Star, Inc., OR, USA).

### Sorting of CD11b^+^Gr-1^+^ Cells and Adoptive Transfer Into Mice With Established EAE

Spleen mononuclear cells from AAV-IL-27 or AAV-ctrl virus-treated mice (with or without EAE) were stained for CD11b and Gr-1, the CD11b^+^Gr-1^+^ cells were then sorted using the Moflo XDP sorter (Beckman Coulter, Indianapolis, IN, USA). To treat mice with EAE using CD11b^+^Gr-1^+^ myeloid cells, we first established EAE in C57BL6 mice, on day 10 post-immunization, mice were treated with AAV-IL-27 or AAV-ctrl virus as described above. Fourteen days after AAV treatment, mice were sacrificed and CD11b^+^Gr-1^+^ myeloid cells were sorted from spleens and were injected i.v. into mice with established EAE (1 million cells/per mouse; day 10 post EAE induction). The mice were observed for EAE development.

### Real-Time PCR

Quantitative real-time PCR was performed using an ABI 7900-HT sequence system (PE Applied Biosystems) using previously determined conditions (33). The following primers were used for amplifying specific genes: actin: 5′-GAG ACC TTC AAC ACC CCA GC-3′ (forward) and 5′-ATG TCA CGC ACG ATT TCC C-3′ (reverse); IL-1b: 5′-CCA CCT CAA TGG ACA GAA TAT CA-3′ (forward) and 5′-CCC AAG GCC ACA GGT ATT T-3′ (reverse); IL-6: 5′-CCA GAG TCC TTC AGA GAG ATA CA-3′ (forward) and 5′-AAT TGG ATG GTC TTG GTC CTT AG-3′ (reverse); IL-12a: 5′-GAC CAA ACC AGC ACA TTG AAG-3′ (forward) and 5′-CTC CCT CTT GTT GTG GAA GAA-3′ (reverse); IL-17a: 5′-CGC AAT GAA GAC CCT GAT AGA T-3′ (forward) and 5′-CTC TTG CTG GAT GAG AAC AGA A-3′ (reverse); IL-23p19: 5′-CCA GCG GGA CAT ATG AAT CTA C-3′ (forward) and 5′-TGT GGG TCA CAA CCA TCT TC-3′ (reverse); IL-10: 5′-ACA GCC GGG AAG ACA ATA AC-3′ (forward) and 5′-CAG CTG GTC CTT TGT TTG AA-3′ (reverse); IFNg: 5′-AGC TCT TCC TCA TGG CTG TT-3′ (forward) and 5′-TTT GCC AGT TCC TCC AGA TA-3′ (reverse); TNFa: 5′-ATG AGA AGT TCC CAA ATG GC-3′ (forward) and 5′-CTC CAC TTG GTG GTT TGC TA-3′ (reverse); GM-CSF: 5′-CTG CGT AAT GAG CCA GGA AC-3′ (forward) and 5′-GTT TGT CTT CCG CTG TCC AA-3′ (reverse); S100A8: 5′-GTC CTC AGT TTG TGC AGA ATA TAA A-3′ (forward) and 5′-TAT CAC CAT CGC AAG GAA CTC-3′ (reverse); S100A9: 5′-GCA CAG TTG GCA ACC TTT ATG-3′ (forward) and 5′-CCA TCA GCA TCA TAC ACT CCT C-3′ (reverse). Each sample (RNA purified from sorted CD11b^+^Gr1^+^ myeloid cells or spinal cords) was assayed in triplicate, and the experiments were repeated two to three times. The relative gene expression was determined using the comparative method (2^−ΔΔCt^).

### Statistics

Data were expressed as means of individual determinations ±SE. Two-tailed Student’s *t*-test or one way ANOVA was used for statistical analyses.

## Results

### Systemic Delivery of IL-27 by AAV Virus Inhibits Th17 Responses and Prevents EAE Development

**Figure 1:**
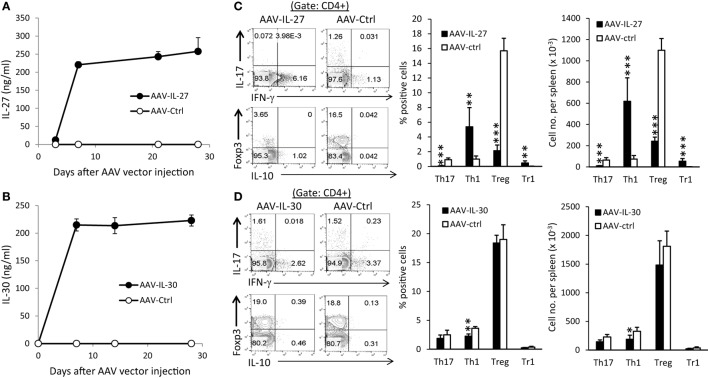
Adeno-associated viral vector (AAV) mediated delivery of interleukin-27 (IL-27)/IL-30 and their impacts on T cell subsets. C57BL6 mice (*n* = 5/group) were treated with AAV-IL-27, AAV-IL-30, or AAV-ctrl virus i.m. at a dose of 2 × 10^11^ DRP/mouse. IL-27 **(A)** or IL-30 **(B)** levels in blood were detected by ELISA. Two weeks after AAV injection, mice (*n* = 5/group) were sacrificed and CD4^+^ T cells in spleens from AAV-IL-27 **(C)** or AAV-IL-30 **(D)** treated mice were analyzed and quantified by flow cytometry. Data shown represent two **(A,B)** and three **(C,D)** experiments with similar results. Statistical analysis was performed using the unpaired Student’s *t*-test. *P < 0.05; **P < 0.01; ***P < 0.001.

To determine if IL-27 or IL-30 can be used as a potential therapeutic for autoimmune diseases, we generated recombinant adeno-associated virus that express IL-27 (AAV-IL-27) or IL-30 (AAV-IL-30) and the control AAV virus (AAV-ctrl). Intramuscular injection (i.m.) of 2 × 10^11^ DRP/mouse of AAV-IL-27 or AAV-IL-30 achieved high and stable IL-27 (Figure 1A) or IL-30 (Figure 1B) production in the peripheral blood of mice. AAV-IL-27 treatment significantly enhanced Th1 response and slightly induced T cell production of IL-10, while reduced the frequencies of Th17 and Treg cells in spleens (Figure 1C). By contrast, AAV-IL-30 treatment slightly inhibited Th1 response but failed to affect the frequencies of Th17/Treg cells and T cell production of IL-10 (Figure 1D).

**Figure 2:**
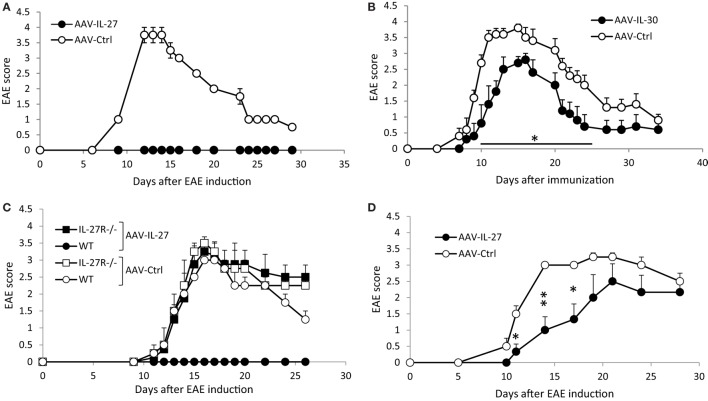
The effects of adeno-associated viral vector (AAV)-delivered interleukin-27 (IL-27) or IL-30 in the prevention of experimental autoimmune encephalomyelitis (EAE) development. C57BL6 mice **(A,B)**, IL-27Rα^−/−^ **(C)**, or 2D2 TCR transgenic mice **(D)** were treated with AAV-IL-27, AAV-IL-30, or AAV-ctrl virus i.m. at a dose of 2 × 10^11^ DRP/mouse. One week later, EAE was induced in the AAV-treated mice. Five mice per group were used in these experiments. Data shown represent two **(C,D)** to three **(A,B)** experiments with similar results. EAE scores are expressed as means ± SD. Statistical analysis was performed using the unpaired Student’s *t*-test. *P < 0.05; **P < 0.01.

To determine if AAV-delivered IL-27 or IL-30 could block EAE development, we injected AAV-IL-27, AAV-IL-30, or AAV-ctrl virus into C57BL6 mice, 1 week later mice were immunized with MOG35-55/CFA and pertussis toxin. In AAV-ctrl virus-treated mice, EAE symptoms developed, with first symptoms showed up on day 10, while disease progressed to peak around days 14–17, then the EAE symptoms went down but maintained at a lower level for a long time (Figures 2A,B). While a single injection of AAV-IL-27 completely prevented EAE development in C57BL6 mice (Figure 2A), a single dose of AAV-IL-30 only slightly inhibited EAE development (Figure 2B). AAV-IL-27 treatment failed to prevent EAE in IL-27Rα^−/−^ mice, suggesting that AAV-IL-27 acts through IL-27 receptor (Figure 2C). 2D2 TCR transgenic mice develop progressive EAE symptoms upon immunization, presumably due to the activation of overwhelming numbers of myelin-specific T cells. We therefore tested if EAE in 2D2 mice could be prevented by AAV-IL-27 treatment. As shown in Figure 2D, AAV-IL-27 administration slightly delayed the onset of EAE symptoms, but significantly inhibited the EAE symptoms in 2D2 mice.

**Figure 3:**
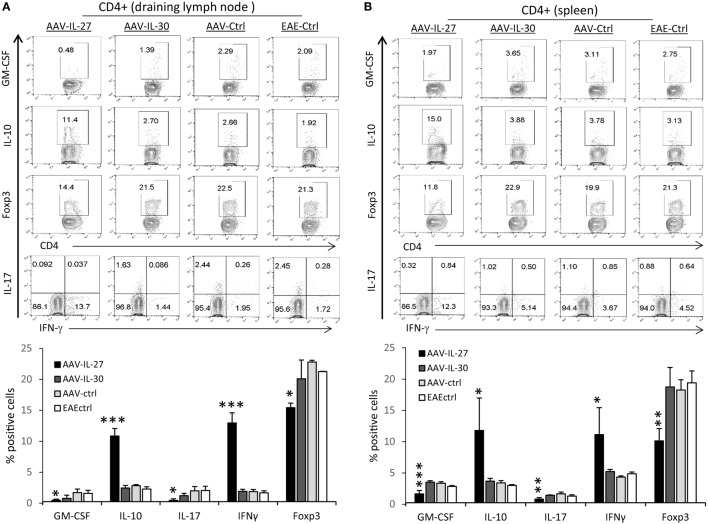
T cell subsets in mice whose experimental autoimmune encephalomyelitis (EAE) development was prevented by adeno-associated viral vector (AAV)-delivered interleukin-27 (IL-27). C57BL6 mice (*n* = 5/group) were treated with AAV-IL-27, AAV-IL-30, and AAV-ctrl virus i.m. at a dose of 2 × 10^11^ DRP/mouse or left untreated. One week later, the mice were induced for EAE and sacrificed 2 weeks after EAE induction. T cell subsets in draining lymph nodes **(A)** and spleens **(B)** were analyzed by flow cytometry. Data were summarized and presented in the lower panels. *P < 0.05; **P < 0.01; ***P < 0.001 by one way ANOVA. Data shown represent two experiments with similar results.

To determine if AAV-IL-27 prevented EAE development by altering T cell responses, we analyzed T cell subsets in the draining lymph nodes (DLNs) and spleens from AAV-IL-27-treated mice and controls. As shown in Figure 3, we found that the CD4^+^ T cells from the immune lymph nodes (Figure 3A) and spleens (Figure 3B) of AAV-IL-27-treated mice had significantly decreased GM-CSF, IL-17, and Foxp3 expressing subsets, while IL-10 and IFN-γ producing subsets increased compared with CD4^+^ T cells from AAV-IL-30-treated or other control groups of mice.

### IL-10 and PD-L1 Independent EAE Inhibition in AAV-IL-27-Treated Mice

**Figure 4:**
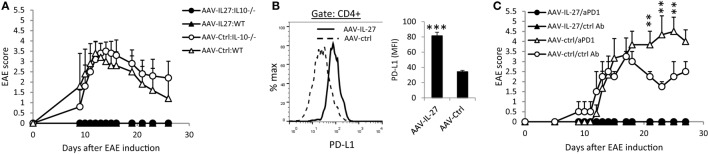
The role of IL-10 and PD-1 blockade in adeno-associated viral vector (AAV)-interleukin-27 (IL-27) mediated prevention of experimental autoimmune encephalomyelitis (EAE). **(A)** WT or IL-10^−/−^ mice were injected with AAV-IL-27 or AAV-ctrl virus i.m. at a dose of 2 × 10^11^ DRP/mouse. One week later, EAE was induced in the AAV virus-treated mice. Five mice per group were used in this experiment, and data shown represent two experiments with similar results. EAE scores are expressed as means ± SD. **(B)** AAV-IL-27 treatment induced T cell expression of PD-L1. Representative FACS plot (left panel) and summary (right panel) of PD-L1 expression on spleen CD4^+^ T cells is shown. Three to four mice per group were included in this experiment, and data represent five experiments with similar results. Statistical analysis was performed using the unpaired Student’s *t*-test. ****P < 0.0001. **(C)** PD-1 blockade failed to break T cell tolerance in AAV-IL-27-treated mice. C57BL6 mice (*n* = 4/group) were first treated with AAV-IL-27 or AAV-ctrl virus followed by EAE induction 1 week later. On days 5, 9, 13, and 17 after EAE induction, mice were also treated with 300 μg/mouse of anti-PD-1 (RMP1-14) or an isotype-matched control antibody (2A3) i.p. EAE scores are expressed as means ± SD. Data shown represent three experiments with similar results. **P < 0.01 by Student’s *t*-test.

Since we detected increased numbers of IL-10 producing Th cells in AAV-IL-27-treated mice, we tested if AAV-IL-27 mediated inhibition of EAE *via* induction of IL-10. C57BL6 or IL-10^−/−^C57BL6 mice were treated with AAV-IL-27 or AAV-ctrl virus, followed by induction of EAE *via* active immunization. While both WT and IL-10^−/−^ mice treated with AAV-ctrl virus developed severe EAE symptoms (Figure 4A), both mice treated with AAV-IL-27 failed to develop EAE. Thus, AAV-IL-27-induced IL-10 production by T cells is insufficient to inhibit T cell-mediated EAE.

Since IL-27 was shown to induce T cell expression of PD-L1, which contributed to T cell tolerance in the EAE model (10), we tested if AAV-IL-27 induced T cell tolerance through induction of PD-L1. As shown in Figure 4B, we found that treatment with AAV-IL-27, but not AAV-Ctrl virus indeed induced significant expression of PD-L1 in T cells. To determine if PD-L1-PD-1 interaction among T cells mediated their tolerance, C57BL6 mice were first treated with AAV-IL-27 or AAV-ctrl virus followed by EAE induction 1 week later. On days 5, 9, 13, and 17 after EAE induction, mice receiving AAV-IL-27 treatment were also treated with 300 μg/mouse of anti-PD-1 or an isotype-matched control antibody i.p. As shown in Figure 4C, while mice treated with AAV-ctrl virus and control antibody exhibited EAE symptoms by day 10 and reached peak disease by day 17, mice treated with AAV-ctrl virus and anti-PD-1 developed more severe EAE, consistent with the known functions of PD-1 blockade in EAE development (34). However, mice treated with AAV-IL-27 + ctrl antibody or AAV-IL-27 + anti-PD-1 showed no EAE symptoms (Figure 4C). Thus, blockade of PD-L1-PD-1 interaction failed to reverse T cell tolerance induced by AAV-IL-27 treatment.

### AAV-IL-27 Treatment Does Not Inhibit Established Th17 Responses and EAE

**Figure 5:**
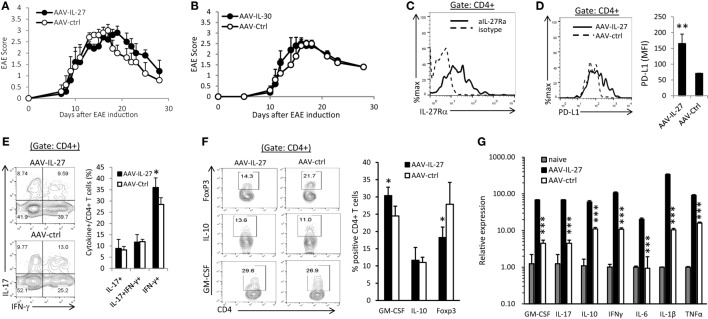
Therapeutic effect of adeno-associated viral vector (AAV)-interleukin-27 (IL-27) or AAV-IL-30 on established experimental autoimmune encephalomyelitis (EAE). C57BL6 mice were immunized with MOG peptide/CFA and pertussis toxin. Ten days after immunization, when the first symptoms of EAE appeared, mice (*n* = 5/group) were treated with AAV-IL-27 **(A)** or AAV-IL-30 **(B)** virus i.m. The mice were then evaluated for the development of EAE signs. EAE scores are expressed as means ± SD. Data shown represent three experiments with similar results. On day 29 after EAE induction, the mice were sacrificed and the expression of IL-27Rα **(C)**, PD-L1 **(D)**, IFN-γ/IL-17A **(E)**, and FoxP3/IL-10/GM-CSF **(F)** in the central nervous system-infiltrating CD4^+^ T cells were analyzed by flow cytometry. Data shown represent two to three experiments with similar results. Statistical analysis was performed using the unpaired Student’s *t*-test. *P < 0.05; **P < 0.01. **(G)** The expression of cytokine genes in the spinal cords of AAV-IL-27 or AAV-ctrl virus-treated mice with established EAE (*n* = 4/group) were assessed by qPCR. Data shown represent two experiments with similar results. ***P < 0.001 by Student’s *t*-test.

To determine if AAV-IL-27 treatment could reverse ongoing inflammation in the CNS, C57BL6 mice were immunized with MOG peptide/CFA and pertussis toxin. Ten days after immunization, when the first symptoms of EAE appeared, mice were treated with AAV-IL-27 or AAV-Ctrl virus i.m. As shown in Figure 5A, AAV-IL-27 treatment at day 10 after EAE induction failed to inhibit EAE development. Similarly, we found that treatment of mice on day 10 after EAE induction with AAV-IL-30 also had no effect on EAE development (Figure 5B). One potential explanation for failure of inhibiting EAE development could be due to lack of IL-27 receptor expression in the CNS-infiltrating CD4^+^ T cells. However, we found high levels of IL-27Rα expression in the CNS-infiltrating CD4^+^ T cells (Figure 5C). Moreover, we found that AAV-IL-27 treatment induced PD-L1 expression in CNS-infiltrating CD4^+^ T cells (Figure 5D) and enhanced Th1 responses without significantly affecting Th17 responses in the CNS (Figure 5E). AAV-IL-27-treatment also significantly inhibited Treg subset without significantly affecting Tr1 subset in the CNS (Figure 5F). GM-CSF-producing CD4^+^ T cells were found to be increased in the CNS of AAV-IL-27 treated mice (Figure 5F). Strikingly, we found that AAV-IL-27 treatment of mice with ongoing EAE upregulated many cytokine genes including GM-CSF, IL-17, IL-10, IFN-γ, IL-6, IL-1β, and TNF-α in the CNS (Figure 5G).

### AAV-Mediated Delivery of IL-27 Induces the Expansion of CD11b^+^Gr1^+^ Myeloid Cells

**Figure 6:**
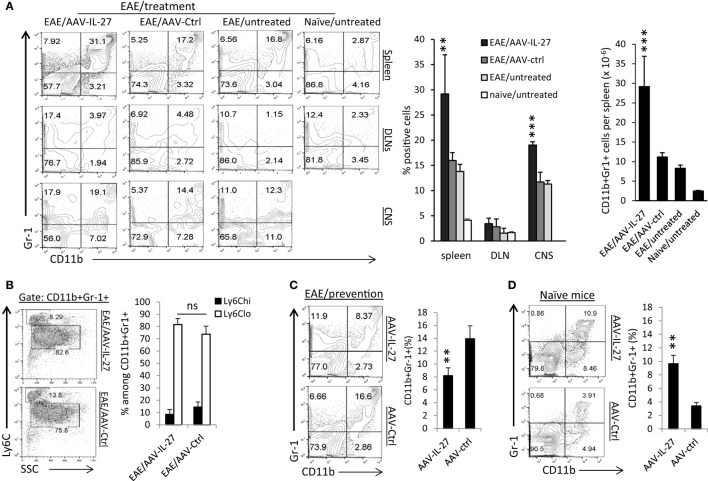
The effects of adeno-associated viral vector (AAV)-delivered interleukin-27 (IL-27) on the expansion of CD11b^+^Gr-1^+^ myeloid cells. **(A)** C57BL6 mice were immunized with MOG peptide/CFA and pertussis toxin. Ten days after immunization, when the first symptoms of experimental autoimmune encephalomyelitis (EAE) appeared, mice (*n* = 5/group) were treated with AAV-IL-27 or AAV-ctrl virus i.m. or left untreated. By day 30 after EAE induction, mice were sacrificed, and myeloid cells in spleens (upper panel), draining lymph nodes (DLNs; middle panel) and spinal cords [central nervous system (CNS)] were analyzed by flow cytometry. Percentages and absolute numbers (spleen) of CD11b^+^Gr-1^+^ myeloid cells were also quantified (right panel). Data shown represent two experiments with similar results. **P < 0.01; ***P < 0.001 by one way ANOVA test. **(B)** Subsets of myeloid cells in AAV-IL-27 or AAV-ctrl virus-treated mice with EAE. Mice (*n* = 6–7/group) were induced for EAE and treated as described above. By day 30 after EAE induction, mice were sacrificed, and spleen cells were stained for CD11b, Gr-1, and Ly6C, and were analyzed by flow cytometry. Statistical analysis was performed using the unpaired Student’s *t*-test. *P < 0.05. Data shown represent three experiments with similar results. **(C)** C57BL6 mice (*n* = 5/group) were injected with AAV-IL-27 or AAV-ctrl vectors i.m. at a dose of 2 × 10^11^ DRP/mouse. One week later, mice were induced for EAE. Two weeks after EAE induction, mice were sacrificed and spleen cells were analyzed for the expansion of CD11b^+^Gr-1^+^ myeloid cells by flow cytometry. **P < 0.01 by Student’s *t*-test. Data shown represent two experiments with similar results. **(D)** C57BL6 mice (*n* = 5/group) were injected with AAV-IL-27 or AAV-ctrl virus i.m. at a dose of 2 × 10^11^ DRP/mouse. Two weeks later, mice were sacrificed and spleen cells were analyzed for the expansion of CD11b^+^Gr-1^+^ myeloid cells by flow cytometry. **P < 0.01 by Student’s *t*-test. Data shown represent five experiments with similar results.

Significant induction of cytokines such as IL-6, IL-1β, and TNF-α in the CNS suggests that AAV-IL-27 treatment may have significant impacts on myeloid cells. Indeed, through the analysis of the myeloid compartment in the peripheral lymphoid organs and CNS, we found that CD11b^+^Gr1^+^ myeloid cells were significantly increased in the spleens and CNS of mice with EAE, and AAV-IL-27 treatment further expanded those cells (Figure 6A). The impact of AAV-IL-27 on this population of cells was dramatic, as in the spleen, CD11b^+^Gr-1^+^ myeloid cells expanded about threefold compared to mice with untreated EAE (Figure 6A, right panel). Notably, we did not find expansion of CD11b^+^Gr-1^+^ myeloid cells in DLNs (Figure 6A). The expanded CD11b^+^Gr-1^+^ myeloid cells were mainly of the Ly6C^low^ subtype, and subtypes were not significantly different between AAV-IL-27 and AAV-ctrl-treated mice (Figure 6B). While we observed a major expansion of CD11b^+^Gr-1^+^ myeloid cells in mice with EAE that received AAV-IL-27 therapy at disease onset, in the EAE prevention model, AAV-IL-27-treated mice had much lower numbers of CD11b^+^Gr-1^+^ myeloid cells compared with AAV-ctrl-treated mice that developed EAE (Figure 6C). These results suggest that CD11b^+^Gr-1^+^ myeloid cells are mainly associated with disease activity. To determine if AAV-IL-27 therapy directly induce expansion of CD11b^+^Gr-1^+^ myeloid cells, we injected AAV-IL-27 or AAV-ctrl virus into naïve C57BL/6 mice and found that AAV-IL-27 treatment could significantly induce expansion of CD11b^+^Gr-1^+^ myeloid cells in naïve mice in the absence of EAE (Figure 6D). These data together suggest that IL-27 alone could induce expansion of CD11b^+^Gr-1^+^ myeloid cells, and in the presence of active EAE the expansion of these myeloid cells become more robust.

**Figure 7:**
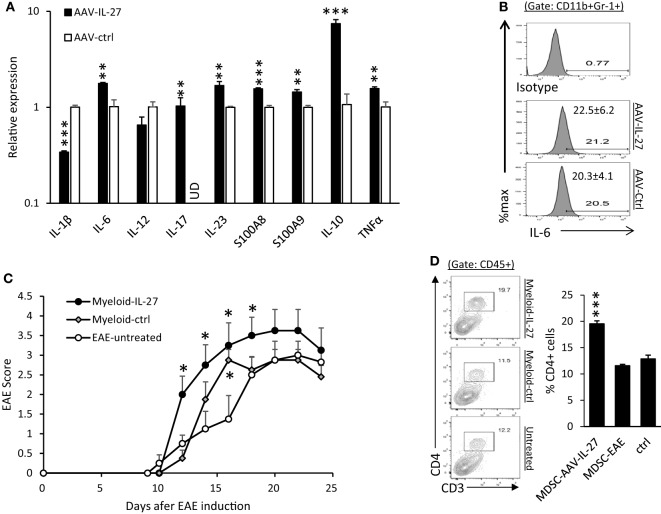
CD11b^+^Gr-1^+^ myeloid cells from adeno-associated viral vector (AAV)-interleukin-27 (IL-27)-treated mice enhance experimental autoimmune encephalomyelitis (EAE) development. **(A)** Myeloid cells from AAV-IL-27-treated mice express more inflammatory cytokine genes. CD11b^+^Gr-1^+^ myeloid cells were FACS sorted from spleens of AAV-IL-27 and AAV-ctrl treated mice. The expression of cytokine genes was assessed by qPCR. The sorted cells from each treatment group (*n* = 3) were mixed, and results were expressed as mean ± SD from triplicates. ud, un detectable. Data shown represent two experiments with similar results. **(B)** Intracellular staining and flow cytometry were used for detecting IL-6 in myeloid cells from AAV-IL-27-treated (*n* = 7) or AAV-ctrl-treated (*n* = 6) mice. Numbers represent mean ± SD of IL-6^+^ cells among the CD11b^+^Gr1^+^ population. Data shown represent two experiments with similar results. **(C)** Adoptive transfer of AAV-IL-27-treated CD11b^+^Gr-1^+^ myeloid cells enhances EAE development. CD11b^+^Gr-1^+^ myeloid cells were FACS-purified from AAV-IL-27 or AAV-ctrl virus-treated mice with EAE and were injected i.v. into mice (1 million cells/per mouse) on day 10 post EAE induction. The mice (*n* = 4/group) were observed for EAE development. *P < 0.05 by one way ANOVA. Data shown represent two experiments with similar results. **(D)** CD4^+^ T cell expansion was observed in the central nervous system (CNS) of mice receiving AAV-IL-27-treated CD11b^+^Gr-1^+^ myeloid cells. Mice indicated in **(D)** were sacrificed on day 24 and flow cytometry was used to analyze the CNS-infiltrating leukocytes. ***P < 0.001 by one way ANOVA.

CD11b^+^Gr-1^+^ myeloid cells have previously been shown to inhibit or enhance EAE development (35, 36). We purified CD11b^+^Gr-1^+^ myeloid cells from the spleens of AAV-IL-27 and AAV-ctrl virus-treated mice by FACS-based sorting, and analyzed their expression of cytokine genes. As shown in Figure 7A, we found that CD11b^+^Gr-1^+^ myeloid cells from AAV-IL-27-treated mice had increased expression of IL-6, IL-17, IL-23, S100A8, A100A9, IL-10, and TNF-α genes. IL-1β expression was decreased compared with myeloid cells from AAV-ctrl treated mice, but remained readily detectable (at 22 cycles by qPCR).

Using flow cytometry analysis, we found that IL-6 protein was readily detectable in CD11b^+^Gr-1^+^ myeloid cells from both AAV-IL-27 and AAV-ctrl treated EAE mice (Figure 7B). To test if IL-27-expanded myeloid cells affect EAE development, CD11b^+^Gr-1^+^ myeloid cells were FACS-purified from AAV-IL-27 or AAV-ctrl virus-treated mice with EAE and were injected i.v. into mice on day 10 post EAE induction. We found that CD11b^+^Gr-1^+^ myeloid cells from AAV-IL-27-treated mice more significantly enhanced EAE development (Figure 7C). Consistent with disease severity, we found that more CD4^+^ T cells infiltrated into the CNS of mice receiving AAV-IL-27-expanded myeloid cells (Figure 7D).

## Discussion

In this study, we have evaluated the efficacy of AAV-delivered IL-27 (AAV-IL-27) and IL-30 (AAV-IL-30) in a murine model of MS. We found that one single administration of AAV-IL-27 completely prevented autoimmune encephalomyelitis, while significant, but incomplete protection was observed in AAV-IL-30-treated mice. AAV-IL-27 treatment inhibited Th17 responses and induced multiple inhibitory pathways in T cells. Strikingly, we found that mice with established EAE was completely resistant to AAV-IL-27 or AAV-IL-30 treatment, and AAV-IL-27 treatment induced the expansion of CD11b^+^Gr-1^+^ myeloid cells that could produce multiple cytokines including Th17-promoting cytokines.

The complete prevention of EAE development in C57BL6 mice by AAV-IL-27 suggests potent protective mechanisms are activated. Indeed, we observed that AAV-delivered IL-27 inhibited the priming of Th17 cells, and induced T cell expression of IL-10 and PD-L1. Inhibition of Th17 response is consistent with previous studies (37, 38) using IL-27 as therapeutic, suggesting that AAV-IL-27-mediated inhibition of Th17 response contributes to the prevention of EAE. We also observed that AAV-IL-27 treatment inhibited the frequencies of GM-CSF-producing T cells in peripheral lymphoid organs in EAE protected mice, which is consistent with the known function of IL-27 in inhibiting GM-CSF production by T cells (17, 24). Although high frequencies of IL-10-producing T cells were induced, our results suggest that AAV-IL-27-induced IL-10 production by T cells is not responsible for induction of T cell tolerance, since AAV-IL-27-treatment induced complete protection of EAE development in IL-10-deficient mice (Figure 4). IL-27-mediated PD-L1 expression in T cells has been shown to be sufficient for inducing T cell tolerance in a mouse model of human MS (10). In this study, we found that PD-L1 was induced in CD4^+^ T cells in the peripheral lymphoid organs (Figure 4) and in the CNS (Figure 5D). However, despite the ability to enhance EAE development in AAV-ctrl treated mice, anti-PD-1 antibody treatment failed to break T cell tolerance induced by AAV-IL-27 (Figure 4C). Thus, PD-L1 expression in T cells is not solely responsible for AAV-IL-27-mediated blockade of EAE development.

Interleukin-27 has multi-faceted roles in T cell responses. While IL-27 has been shown to inhibit Th1 responses (39), majorities of studies have shown that IL-27 enhances Th1 responses by activating Stat1–T-bet axis (40–42). IL-27 has been shown controversial roles in Tregs (43–47), but in IL-27 transgenic mice, Treg cells are deleted (45). In this study, we found that AAV-IL-27 treatment enhanced Th1 responses and downregulated Treg frequencies. However, increased Th1 responses and reduced Treg cells did not reverse AAV-IL-27-mediated EAE protection, suggesting that IL-27-induced inhibition of Th17 priming, and may be together with the activation of other inhibitory pathways are sufficient to prevent EAE development.

A striking finding in this study is that established EAE was resistant to AAV-IL-27 treatment, and in the CNS of AAV-IL-27-treated mice, CD4^+^ T cell production of key inflammatory cytokines such as IL-17 and GM-CSF were not affected or even elevated (Figures 5E,F). Lack of suppression of ongoing EAE by AAV-IL-27 could be due to the blood–brain barrier (BBB) prevented IL-27 access to the CNS or due to unresponsiveness of CNS-infiltrating T cells. However, these possibilities are highly unlikely. It is known that BBB is wide open during the CNS inflammation (48, 49), and T cells in the CNS of mice with EAE expressed high levels of IL-27 receptor (Figure 5C). It is also unlikely that lack of suppression of ongoing inflammation is due to delayed production of IL-27 by AAV virus, since we observed that AAV-mediated IL-27 production was efficient (by day 3 > 10 ng/mL of IL-27 can be detected in blood). More importantly, we found clear evidence that the CNS T cells from AAV-IL-27-treated mice were stimulated by IL-27, which is reflected by induction of PD-L1 expression, increased Th1 and decreased Treg responses (Figure 5). Our results presented in Figures 6 and 7 suggest that the resistance of ongoing Th17-mediated CNS inflammation to IL-27 therapy could be due to the expansion of CD11b^+^Gr-1^+^ cells. It is well established that CD11b^+^Gr-1^+^ cells expand during EAE development (35, 36). However, the role of this population of cells in EAE development is not clearly understood. Adoptive transfer experiment showed that these cells inhibited EAE development (35). However, other study clearly showed that these cells promoted Th17 responses *via* production of IL-1β, and depletion of this population of cells ameliorated EAE development (36). In this study, we found that mice with established EAE had significant expansion of CD11b^+^Gr-1^+^ cells, and AAV-IL-27 treatment further expanded these cells. Moreover, we found that AAV-IL-27 treatment could directly induce the expansion of CD11b^+^Gr-1^+^ cells (Figure 6D), and adoptive transfer of CD11b^+^Gr-1^+^ cells from AAV-IL-27-treated mice enhanced EAE development (Figure 7C). Thus, AAV-IL-27 therapy-induced expansion of CD11b^+^Gr-1^+^ cells enhances EAE development.

Expansion of CD11b^+^Gr-1^+^ myeloid cells mainly occurred in the spleens and CNS but not in DLNs, suggesting that these cells do not regulate T cell priming but mainly act at the effector phase of EAE development. This observation partially explains why EAE development was completely prevented despite some CD11b^+^Gr-1^+^ myeloid cell expansion was observed (Figure 6C). The cytokine profiling of IL-27-expanded CD11b^+^Gr-1^+^ myeloid cells provides an explanation for why these cells enhance EAE development or confer resistance to IL-27 therapy. AAV-IL-27-induced myeloid cells express multiple Th17-promoting cytokines including IL-1β, IL-6, IL-17 and IL-23. IL-1β, IL-6, and IL-23 have been well established as key cytokines for Th17 cell induction/amplification and EAE development (23, 36, 50, 51). Although we observed reduced IL-1β expression in FACS-sorted, IL-27-expanded CD11b^+^Gr-1^+^ myeloid cells (Figure 7A; reduced but still readily detectable by qPCR at 22 cycles), the overall expression of IL-1β increased in the CNS of AAV-IL-27-treated mice (Figure 5G), suggesting that more CD11b^+^Gr-1^+^ myeloid cells accumulated in the CNS and served as a major source of IL-1β. AAV-IL-27-induced CD11b^+^Gr-1^+^ myeloid cells also express S100A8/S100A9, which have been implicated in the inflamed CNS of mice with EAE (52) and shown to promote IL-1β and IL-6 production by immune cells (53). Thus, AAV-IL-27 therapy induces key cytokines for Th17 response in these cells, which could amplify the pre-existing Th17 cells during the effector phase of EAE. In addition to pro-inflammatory cytokines, we also observed that IL-27-expanded CD11b^+^Gr-1^+^ myeloid cells expressed high levels of IL-10, which could explain why high levels of pro-inflammatory cytokines detected in the CNS (Figure 5G) did not cause much worse disease (Figure 5A).

Lack of suppression of ongoing autoimmunity by AAV-IL-27 is consistent with the report (54) showing that IL-27 could not inhibit established Th17 responses, but appears to be inconsistent with other reports (9, 13) demonstrating that systemic IL-27 inhibits T cell adoptive transfer EAE. At this stage, we do not know the reason for this inconsistency. AAV-mediated delivery of IL-27 is highly efficient and results in stable and high concentrations in the blood of the treated mice, and this is not easily achievable by systemic injection of IL-27 protein. It is thus necessary to determine if low and high concentrations of IL-27 induce T cell tolerance *via* different mechanisms. On the other hand, since adoptive transfer EAE involves a latent phase before EAE signs appear (55, 56), suggesting that Th17 priming/differentiation *in vivo* is still needed for causing disease after T cell transfer. Thus, the initial stage of T cell adoptive transfer should not be considered as mice having an ongoing disease, and thus it is not surprising to see that IL-27 could suppress adoptive transfer EAE.

Taken together, our study suggests that systemic delivery of IL-27 can efficiently prevent EAE development and the priming of Th17 cells. However, the therapeutic potential of IL-27 may be limited by its failure to shut down established Th17 responses and reverse ongoing inflammation, presumably due to the expansion of CD11b^+^Gr-1^+^ myeloid cells. Moreover, the depletion of Treg cells adds additional risk for IL-27-based therapy of autoimmune diseases like MS.

## Ethics Statement

This study was approved by the Institutional Animal Care and Use Committee (IACUC) of The Ohio State University (Protocol#: 2008A0093R2 and R3).

## Author Contributions

JZ, J-QL, ZL, and LW performed most of the experiments; MS performed IL-27 ELISA; JZ and JD helped for the production of AAV viruses. X-FB designed all experiments, performed data analyses, and wrote the manuscript.

## Conflict of Interest Statement

The authors declare that the research was conducted in the absence of any commercial or financial relationships that could be construed as a potential conflict of interest.
